# Targeting mitochondrial oxidative phosphorylation: lessons, advantages, and opportunities

**DOI:** 10.1038/s41416-023-02394-9

**Published:** 2023-08-10

**Authors:** Nicole D. Machado, Lisa C. Heather, Adrian L. Harris, Geoff S. Higgins

**Affiliations:** 1 Department of Oncology, University of Oxford, Old Road Campus Research Building, Roosevelt Drive, Oxford, United Kingdom; 2 Department of Physiology, Anatomy and Genetics, University of Oxford, Oxford, United Kingdom

**Keywords:** Cancer metabolism, Drug development, Targeted therapies

## Abstract

In light of the disappointing termination of clinical trials with potent complex I inhibitors, such as IACS-010759, justification for oxidative phosphorylation inhibitors and mitochondrial targeting strategies has been called into question. Consideration of these agents’ potency, tissue selectivity and toxicity demonstrate what lessons can be learned from this failure and where new opportunities lie.

The field of cancer metabolism has evolved greatly over the last 100 years, from the seminal discovery of aerobic glycolysis in tumours to more recent evidence that respiration is not inconsequential and is frequently enhanced in cancer. More specifically, as dependencies on oxidative phosphorylation (OXPHOS) appear prevalent in a variety of aggressive cancer types, the development of OXPHOS inhibitors through targeting the electron transport chain has shown promising pre-clinical efficacy [1]. However, termination of clinical trials in oncology utilising electron transport chain complex I selective inhibitors BAY87-2243, ASP4132, and most recently IACS-010759, due to dose-limiting toxicities, has raised questions on the clinical viability of such approaches [2–4].

IACS-010759 was developed as a highly potent complex I inhibitor and demonstrated promising anti-cancer efficacy in multiple animal models within a moderate safety window. However, Yap et al. have carefully reported the results of two phase I clinical trials of IACS-010759 in patients with advanced solid tumours (23 patients), some with OXPHOS sensitive mutations, and refractory acute myeloid leukaemia (AML) (17 patients). IACS-010759 had unacceptable toxicities including elevated blood lactate, lactic acidosis, vomiting, and peripheral neuropathy; resulting in early trial termination [4]. Dose-related toxicities prohibited maintenance of target plasma levels, with only 1 patient achieving a partial response.

The extremely narrow, if any, therapeutic window of IACS-010759, is likely attributed to its highly potent, on-target complex I inhibition, which operates on a nanomolar range in vitro, similar to toxins targeting complex I e.g., rotenone. Excessive inhibition of mitochondrial respiration is clearly associated with a glycolytic shift towards increased lactate production and lactic acidosis, a common adverse event with IACS-010759. However, other less potent OXPHOS inhibitors such as metformin, nitric oxide and arsenic trioxide, have been widely used in clinics for decades for indications outside of oncology and have established safety profiles [2]. Many moderate OXPHOS inhibitors assessed in oncology clinical trials have demonstrated safe administration and tolerability with other therapies [5]. Moderate OXPHOS inhibitor and antimalarial drug, atovaquone, which specifically targets complex III, produced an anti-cancer effect in vitro at clinically achievable plasma concentrations and retrospective analysis in AML patients correlated higher atovaquone dosing with lower relapse rates [6]. Additionally, the metabolic plasticity of cancers may limit the therapeutic window of OXPHOS inhibitors as monotherapies, but their use in combination therapies, specifically to target cancer stem cells and chemotherapy resistant cancers has been evaluated extensively preclinically. Treatment with complex I inhibitor OPB-51602 demonstrated re-sensitisation to tyrosine kinase inhibitors in vitro, and also resulted in significant tumour regression and metabolic responses in patients with secondary resistance to epidermal growth factor receptor inhibitors in a phase 1 trial [7].

The clinical applications of OXPHOS inhibitors can extend beyond anti-proliferative chemotherapies. Tumour hypoxia, which results from an imbalance between oxygen demand and delivery within the chaotic vasculature of solid malignancies, is strongly implicated in radiation therapy resistance. Modest reductions in cellular oxygen consumption, modelled to be approximately 30% can increase local oxygen availability in tumours to alleviate hypoxia and improve radiosensitivity [8]. Reducing cellular oxygen consumption can be achieved through inhibition of the electron transport chain and several moderate OXPHOS inhibitors have demonstrated tumour reoxygenation and metabolic radiosensitization in preclinical models (Fig. 1a), including papaverine, an antispasmodic drug found to also inhibit complex I [9]. Such strategies would require adequate drug levels in tumours and a subsequent hypoxia modifying effect only at the time of radiation and this can be achieved with moderate OXPHOS inhibition. In non-small cell lung cancer (NSCLC) patients, atovaquone delivered at standard oral doses demonstrated significant increases in tumour reoxygenation [10]. This study clearly demonstrated OXPHOS inhibition resulted in a pharmacodynamic effect achieved within a safe clinical window. Clinical trials in NSCLC patients assessing the efficacy of the combination of atovaquone or papaverine with either chemoradiotherapy or stereotactic body radiotherapy are ongoing (NCT04648033, NCT03824327, NCT05136846).

**Fig. 1:**
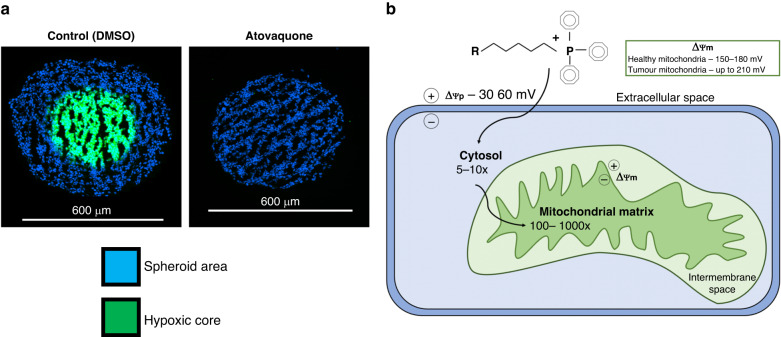
Mitochondrial targeting agents for hypoxia modulation and selective uptake in cancer. **a** The hypoxic core, as visualised by EF5 staining, is completely abolished in HCT116 spheroids following treatment with atovaquone at 30 μM. The scale is 600 μm. **b** Schematic overview of mitochondrial accumulation of triphenylphosphine (TPP+) based therapeutics, where R represents a drug conjugated to TPP+, ΔΨp is plasma membrane potential, and ΔΨm is mitochondria membrane potential.

Increasing the differential biological effect a drug has on its target tissue is another strategy that can improve therapeutic benefit without exacerbating healthy tissue toxicity. Drug design strategies to mitigate this can include the addition of mitochondria targeting moieties, such as triphenylphosphine (TPP+). TPP+ is a lipophilic molecule that possesses delocalised cations enabling its accumulation in the mitochondria matrix according to the charge differential across the plasma and mitochondria membranes – see Fig. 1b [11]. More importantly, the mitochondrial membrane potential has been documented to be substantially higher in tumour cells than normal tissue and is associated with a more invasive cellular phenotype, thus selectively rendering tumour cells more susceptible to drug accumulation with TPP+ conjugates [11]. Gamitrinib, a TPP+ conjugate of a heat shock protein-90 inhibitor, has demonstrated improved pharmacokinetics compared to its parent compound and possesses a safety profile in rats and dogs several fold greater than its effective doses in mice [12]. A phase I clinical trial of gamitrinib in advanced solid tumours is ongoing and will provide insight to how the addition of tumour mitochondria targeting strategies may improve therapeutic outcomes (NCT04827810). In a lung carcinoma model, tumour heterogeneity of TPP+ uptake was also observed, demonstrating that total tumour targeting may not be achieved solely by this strategy [13]. However, metabolic radiosensitizers do not require OXPHOS inhibition in all regions of a tumour to observe hypoxia modulation, indicating that TPP+ based agents can still provide improved safety and therapeutic benefit in this context. Tumour selectivity may also be improved through the use of antibody drug conjugates (ADCs). Several ADCs have been approved and are currently being developed for haematological malignancies, for which OXPHOS dependencies can be frequent [1, 14]. Additionally, ADCs commonly utilise potent drug payloads, which can be leveraged to trial toxic agents originally discarded in early clinical trials or untested as single agents, as seen with the development of Heidelberg Pharma’s alpha amanitin ADC [14]. It will be essential to have pharmacodynamic endpoints in the aforementioned trials, such as metabolic profiles for lactate and mitochondrial effects, repeat biopsies for histology, and imaging scans for hypoxia and its modulation.

While the early termination of IACS-010759 comes as disappointing news in the development of metabolism targeting therapies, there are several ways ahead for selectively targeting mitochondria in cancer. Preclinical toxicology highlights the problems of using different species which have subtle differences in the susceptibility of their mitochondria at a target and tissue level. Considerations for the impact of OXPHOS inhibition on the tumour microenvironment, including proliferation of different types of T-cells, should also be evaluated carefully in the development of new inhibitors. Additional approaches may include organotypic tissue cultures of normal susceptible tissues, tumour organoids, and immunocompetent in vivo models such as genetically engineered mouse models.
